# Does the StartReact Effect Apply to First-Trial Reactive Movements?

**DOI:** 10.1371/journal.pone.0153129

**Published:** 2016-04-14

**Authors:** Katrin Sutter, Jorik Nonnekes, Valeria Dibilio, Alexander C. Geurts, Vivian Weerdesteyn

**Affiliations:** 1 Radboud University Medical Centre, Donders Centre for Neuroscience, Department of Rehabilitation, Nijmegen, The Netherlands; 2 Department GF Ingrassia, Section of Neurosciences, University of Catania, Catania, Italy; 3 Sint Maartenskliniek Research, Nijmegen, The Netherlands; University of Toronto, CANADA

## Abstract

**Introduction:**

StartReact is the acceleration of reaction time by a startling acoustic stimulus (SAS). The SAS is thought to release a pre-prepared motor program. Here, we investigated whether the StartReact effect is applicable to the very first trial in a series of repeated unpractised single-joint movements.

**Methods:**

Twenty healthy young subjects were instructed to perform a rapid ankle dorsiflexion movement in response to an imperative stimulus. Participants were divided in two groups of ten. Both groups performed 17 trials. In one group a SAS (116 dB) was given in the first trial, whereas the other group received a non-startling sound (70 dB) as the first imperative stimulus. In the remaining 16 trials, the SAS was given as the imperative stimulus in 25% of the trials in both groups. The same measurement was repeated one week later, but with the first-trial stimuli counterbalanced between groups.

**Results:**

When a SAS was given in the very first trial, participants had significantly shorter onset latencies compared to first-trial responses to a non-startling stimulus. Succeeding trials were significantly faster compared to the first trial, both for trials with and without a SAS. However, the difference between the first and succeeding trials was significantly larger for responses to a non-startling stimulus compared to responses triggered by a SAS. SAS-induced acceleration in the first trial of the second session was similar to that in succeeding trials of session 1.

**Discussion:**

The present results confirm that the StartReact phenomenon also applies to movements that have not yet been practiced in the experimental context. The excessive SAS-induced acceleration in the very first trial may be due to the absence of integration of novel context-specific information with the existing motor memory for movement execution. Our findings demonstrate that StartReact enables a rapid release of motor programs in the very first trial also without previous practice, which might provide a behavioural advantage in situations that require a rapid response to a potentially threatening environmental stimulus.

## Introduction

Reaction time can be accelerated by a startling acoustic stimulus (SAS), a phenomenon known as StartReact [1,2]. The SAS presumably releases a pre-prepared motor program [3], as evidenced by motor preparation being a prerequisite for the acceleration of SAS-induced reaction times [2]. The significance of this phenomenon for motor control in daily life is poorly understood. A recent review suggested that StartReact provides a behavioral advantage for rapid and task-appropriate responses to potentially threatening stimuli, reminiscent of escape reflexes in invertebrates [4]. Thus, such fast muscular responses could serve as a defensive mechanism in a threatening situation. This interpretation would imply that a startling stimulus should be capable of advancing the release of a prepared movement in the absence of its recent practice in the specific context, provided that the movement itself is fully mastered.

Previous studies have invariably investigated StartReact effects by studying SAS-induced acceleration of movements that had been executed repeatedly in the experimental context prior to the first presentation of the SAS. Hence, it is yet unknown whether movements could be pre-programmed and released by the SAS without previous practice. Alternatively, advance release of a prepared motor program may depend on the preceding—identical—movements, as previous practice strengthens the effective connectivity of the involved motor networks [5]. Under the latter hypothesis, the StartReact effect would be absent when the very first movement is accompanied by a SAS.

We aimed to address this question by evaluating the effect of a SAS versus a non-startling auditory stimulus on reaction times of the very first ankle dorsiflexion movement in an experimental context. In addition, we compared the SAS-induced accelerations (if any) to the StartReact effect after repetitive ankle dorsiflexion movements. Ankle dorsiflexion movements are considered to represent a highly common and fully mastered motor task of daily life (e.g. releasing the gas pedal in response to illuminating brake lights). We reasoned that the presence of substantial SAS-induced acceleration in the first trial would indicate that motor programs can indeed be readily prepared and released, without reliance on the readiness of the involved neural circuits for motor execution by preceding movements. In addition, to identify potential differential effects of the novelty of the experimental context on first-trial reaction times, our study also included a second experimental session, one week after the first. In light of the suggested behavioral significance of the StartReact effect, we expected to see SAS-induced acceleration of reaction times in the first trial of both experimental sessions.

## Materials and Methods

### Participants

Twenty healthy subjects (mean age 27 years, range 23–30 years, 5 men) participated. None of the subjects suffered from any hearing, neurological or motor disorder that could interfere with their performance during the experiments. All participants gave written informed consent prior to the experiment. The study was performed in accordance with local ethical guidelines and was approved by the local ethics committee (medical-ethical committee Arnhem-Nijmegen). The study was conducted in accordance with the Declaration of Helsinki.

### Experimental setup and protocol

Participants were seated in a chair, and performed a forewarned simple reaction task involving ankle dorsiflexion with their dominant foot without target constraints. The participants received verbal instructions on the task and the researcher demonstrated the requested ankle dorsiflexion movement prior to the measurement. Importantly, practice movements or trials were not allowed, the absence of which was closely monitored by the researcher.

An auditory tone (80 dB sound pressure level) was used as a warning signal and participants were instructed to perform a rapid ankle dorsiflexion movement after the second auditory stimulus (i.e., imperative stimulus; 70 dB sound pressure level). Auditory stimuli were presented through headphones. Warning periods (1–3.5 s) and inter-trial periods (6–10 s) were variable. In some trials, the imperative stimulus was replaced by a startling auditory stimulus (SAS; 116 dB sound pressure level). The participants were informed that the sound pressure level of the second auditory stimulus could differ. The SAS was not presented to the participants prior to the reaction time task.

Participants were divided in two groups of ten. Both groups performed 17 trials. In one group, the SAS was given in the first trial, whereas the other group received the non-startling stimulus as their first imperative stimulus. In both groups, a SAS was given as the imperative stimulus in 4 out of the remaining 16 trials. The assessment was repeated one week later, but the presentation of the SAS in the first trial was counterbalanced across the two groups.

### Data collection

Electromyographic (EMG) data were collected from the dominant tibialis anterior and gastrocnemius medialis muscle and right sternocleidomastoid muscle (ZeroWire, Aurion). EMG signals were sampled at 2000 Hz and full-wave rectified and low-pass filtered at 30 Hz (zero-lag, second order Butterworth filter). To determine movement onset, a triaxial accelerometer was placed on top of the dominant foot. Accelerometer signals were sampled at 2000 Hz.

### Data analysis

Two reaction time parameters were assessed: EMG reaction time and accelerometer reaction time. Onset latencies of tibialis anterior and gastrocnemius were determined using a semiautomatic computer algorithm that selected the first instant at which the mean EMG activity exceeded a threshold of 2 SD above the mean background activity, as calculated over a 500 ms period just before the imperative go signal. Onsets were first selected by the computer algorithm, then visually approved and (when necessary) corrected (see method: [6–7]). The onset of foot acceleration was determined in the same manner. Onset latencies were identified for each trial separately. Thereafter, average onset latencies for trials with and without a SAS were calculated over trials 2–17. These trials were termed ‘succeeding trials’.

For each trial in which a SAS was applied, we determined whether a startle reflex occurred. A startle reflex was defined as a short latency response in the SCM muscle rising above baseline level +2SD and starting within 130 ms following the SAS [see method: Nonnekes et al. 2014].

### Statistical analysis

We first verified for the ‘succeeding trials’ a) that the two groups of participants did not differ regarding their onset latencies and b) that the SAS significantly shortened the onset latencies. To this aim, a repeated-measures ANOVA was used, with *SAS (SAS—no SAS)* as within-subjects factor and *Group (group with SAS as first trial—group with nonstartle as first trial)* as between-subjects factor.

We then compared, for session 1, the first-trial onset latencies to those in the succeeding trials. To this aim, a repeated measures ANOVA was used, with *Order* (*first trial—succeeding trials with same stimulus*) as within-subjects factor and *SAS (SAS as first trial—nonstartle as first trial*) as between-subjects factor. A similar analysis was performed to compare the onset latencies in the succeeding trials of session 1 to those in the first trial of session 2. The alpha-level was set at 0.05.

## Results

**Fig 1:**
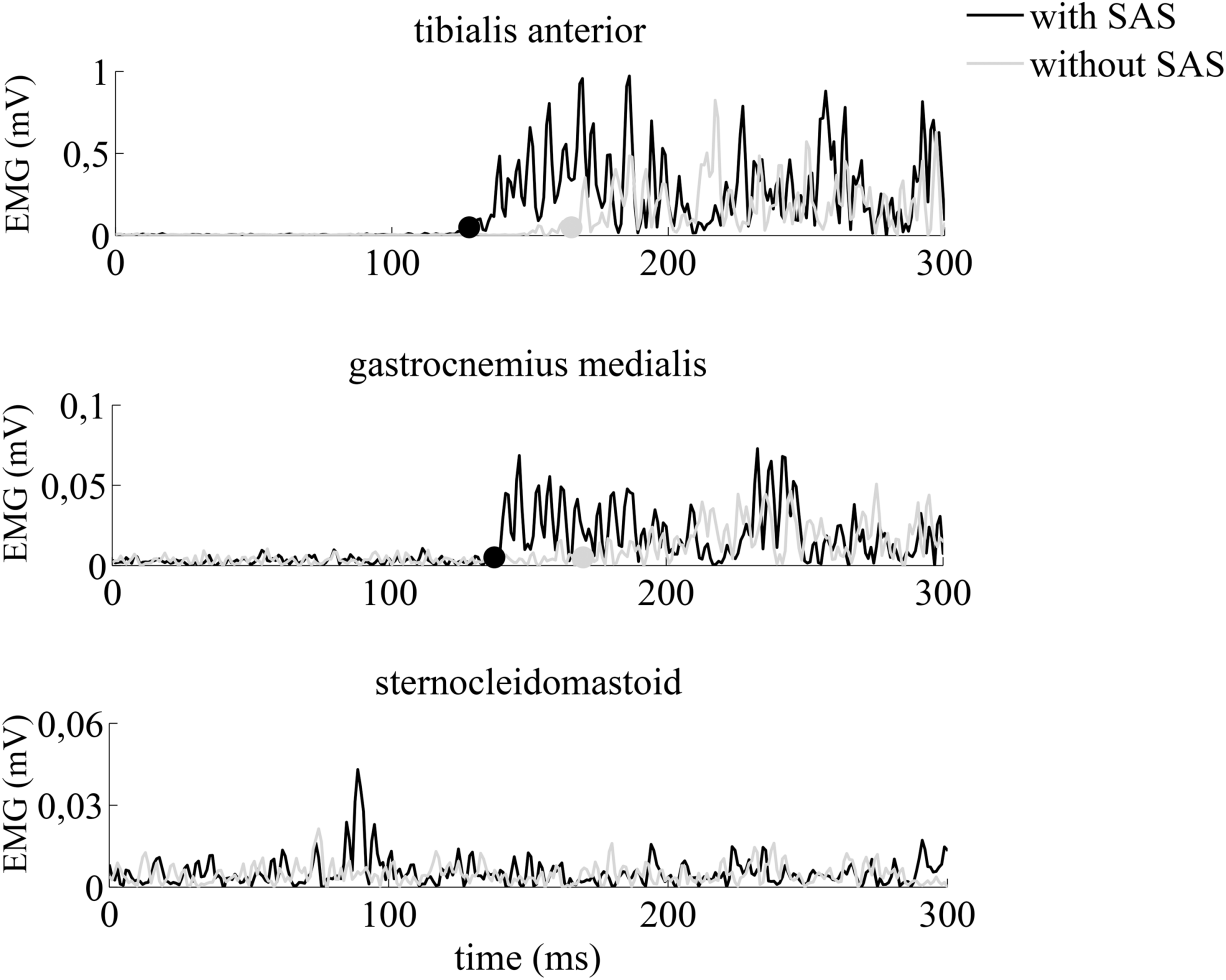
Raw EMG traces of a representative trial during ankle dorsiflexion. Gray lines are trials without a SAS, black lines are trials with a SAS. Determined latencies are presented by a dot.

In all participants we observed a sequential activation of tibialis anterior muscle and gastrocnemius medialis both in trials with and without SAS (see Fig 1). The average interval between tibialis anterior and gastrocnemius medialis onset latencies was 18 ms with SAS and 24 ms without a SAS.

With regard to the onset latencies in the tibialis anterior muscle in the succeeding trials, the two groups of participants indeed did not differ (Group: F_(1,18) =_ 0.046, p = 0.832). The analysis also confirmed that reaction times with a SAS were faster compared to those without a SAS (122±25 ms versus 157±40 ms)(SAS: F_(1,18)_ = 34.150, p<0.001). This pattern of results was also observed in gastrocnemius medialis and in movement onset latencies with similar significance levels for Group and SAS main effects (Group, p>0.90; SAS, p<0.001).

**Fig 2:**
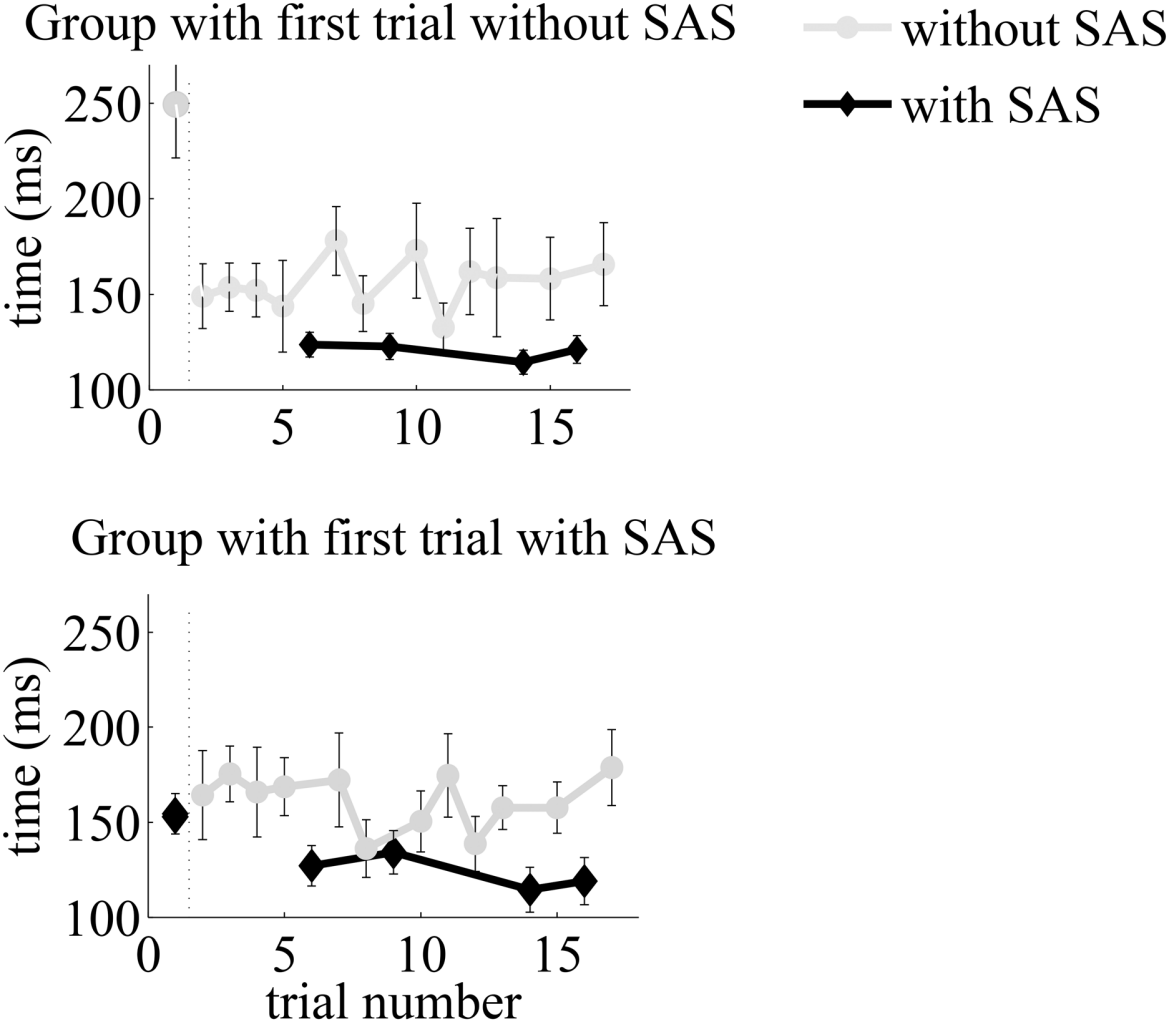
Mean onset latencies per trial during the simple reaction time task with voluntary ankle dorsiflexion. Gray lines are trials without a SAS, black lines are trials with a SAS. The error bars indicate standard errors.

**Fig 3:**
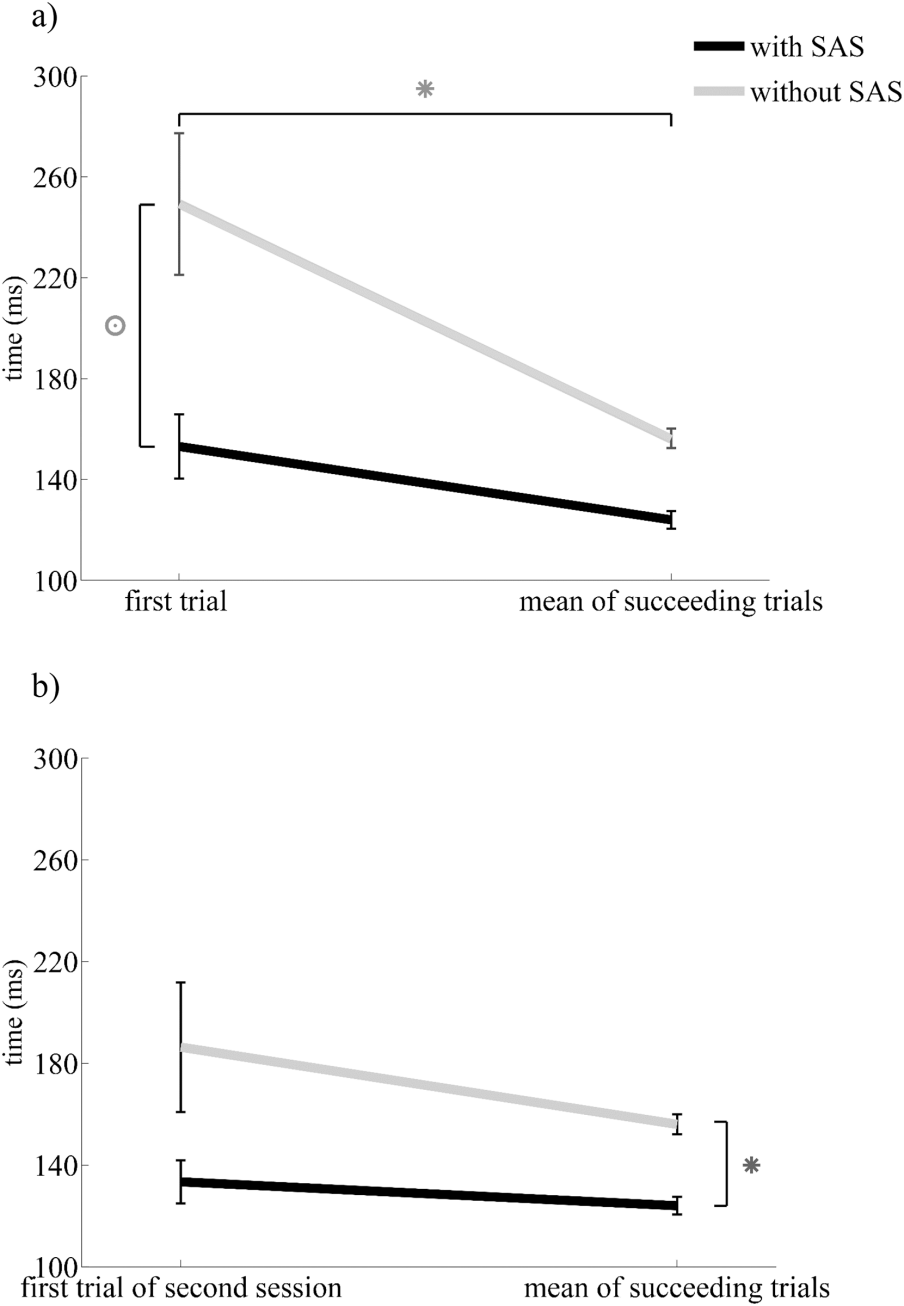
a) Mean onset latencies of tibialis anterior muscle during the simple reaction time task with voluntary ankle dorsiflexion in first trial of first session and the succeeding trials. *Significant differences between first trials and mean of succeeding trials. ס Significant SASxGroup interaction. b) Mean onset latencies of tibialis anterior muscle during the simple reaction time task with voluntary ankle dorsiflexion in succeeding trials of first session and the first trial of second session. *Significant main effect of SAS.

In the first trials with SAS, the onset latency of tibialis anterior activation was shorter than in the first trial without SAS, and this difference was much larger than in the succeeding trials (SASxOrder: F_(1,18)_ = 4.776, p = 0.042, see Figs 2 and 3a). First-trial reaction times with the SAS were on average 96 ms faster than those without (153±34 versus 249±89 ms), whereas the difference was only 33 ms for succeeding trials (123±33 versus 156±42 ms).

Reaction times were significantly longer in the first trial compared to succeeding trials with the same stimulus (Order: F_(1,18)_ = 18.154, p<0.001), but inherent in the above interaction effect, the difference was larger for trials without a SAS (93 ms) than for trials with a SAS (30 ms). Paired t-tests revealed that the differences between first and subsequent trials were significant for both types of stimuli (without SAS t(9) = 3.395, p = 0.008; with SAS t(9) = 3.300, p = 0.009).

A similar pattern of results was observed regarding the onsets of gastrocnemius medialis activity and movement onsets as determined using the accelerometer. The onset latencies in the gastrocnemius medialis muscle were longer in the first trial compared to succeeding trials with the same stimulus (Order: F_(1,18)_ = 14.379, p = 0.001), but the difference between the first and succeeding trials was again larger (on average 72 ms) for trials without a SAS (274±103 ms versus 178±49 ms) than for trials with a SAS (170±38 ms versus 146±31 ms; SASxOrder: F_(1,18)_ = 5.381, p = 0.032).

Movement onsets showed a similar effect of Order (F_(1,18)_ = 14.897, p = 0.001), as well as a 72 ms greater difference between the first and succeeding trials for trials without a SAS (277±103 ms versus 178±46ms) than for trials with a SAS (172±37 ms versus 145±38 ms; SASxOrder: F_(1,18)_ = 4.863, p = 0.041).

In contrast to the greater effects of the SAS in the first trial of session 1, the SAS-induced acceleration in the first trial of session 2 was similar to that in the succeeding trials of session 1 (Fig 3b). This was evidenced by a significant main effect of SAS (F_(1,18)_ = 6.034, p = 0.024) and an absent SASxOrder interaction effect (F_(1,18)_ = 0.499, p = 0.489). In the first trial of session 2, tibialis onset latencies in response to the SAS were 133±27 ms versus 186±80 ms for non-startling stimuli. Onset latencies in the first trial of session 2 were on average 20 ms delayed compared to those in succeeding trials of session 1, yet this difference failed to reach significance (F_(1,18)_ = 4.275, p = 0.053).

Of note, we also observed a pattern of delayed reaction times to non-startling stimuli when they were directly preceded by a SAS trial (Fig 2). Although not formally within the scope of our primary study aim, we conducted an additional statistical test to compare tibialis anterior onset latencies in succeeding trials following a SAS trial to those in succeeding trials following non-SAS trials. These data were non-normally distributed and, therefore, we applied a Wilcoxon signed-rank test. This test indeed demonstrated significantly delayed reaction times in non-SAS trials immediately following a SAS trial (*Z* = -2.427, *p* = 0.015).

### Startle reflexes in sternocleidomastoid muscle

We observed a startle reflex in the sternocleidomastoid (SCM) muscle in 67% of the trials with a SAS. Only one subject did not show an SCM burst in the first trial with SAS. The TA onset latency of this subject was 167ms, which was well within the range of the group at large (153±34 ms). Without this one subject, the average TA onsets for the first trials with a SAS and with SCM burst were 152±35 ms (cf. 122±25 ms for succeeding trials with a SAS). SCM onset latencies did not differ between groups (64±5 ms versus 68±2 ms, p = 0.227) and also did not differ between the first trial with a SAS and the succeeding trials with a SAS (p = 0.436). A within-subjects comparison in participants who had SAS trials both with and without startle reflex in SCM (n = 5), yielded no difference in tibialis anterior onsets in the presence or absence of startle reflexes in SCM (139±37 ms with startle reflex versus 149±30 ms without startle reflex, p = 0.782).

## Discussion

In the present study we investigated whether the StartReact effect is applicable to familiar, yet unpracticed movements. To this aim, we evaluated reaction times of the first voluntary ankle dorsiflexion movement with a SAS or with a non-startling stimulus and compared these to reaction times of the succeeding ankle dorsiflexion movements. When a SAS was given in the very first trial, participants had on average 96 ms faster reaction times compared to first-trial responses to a non-startling stimulus. Succeeding trials were significantly faster compared to the first performed trial, both for trials with and without a SAS. However, the difference between first and succeeding trials was substantially larger for responses to a non-startling stimulus compared to responses triggered by a SAS.

The present study is the first to report that the SAS-induced acceleration is present when a movement is performed without previous practice in the experimental context. There has been one previous account of the presence of a StartReact effect in the first trial of a wrist extension movement when a startling stimulus was applied coincident with the imperative stimulus. In that study, however, practice trials of the wrist movement preceded the actual experiment [8], which were not allowed in the present study. In another study it was found that first-trial postural responses following unexpected external perturbations were greater in amplitude than succeeding trials, presumably due to the startling nature of the perturbation; onset latencies were not compared between first and succeeding trials [9]. Furthermore, as the perturbation direction was unknown to the participants, the response could not be programmed in advance. This may explain why the first-trial response appeared to reflect the superposition of a generalized startle reflex, rather than a startle-induced release of the directionally-appropriate postural response. Indeed, from earlier studies, it is evident that the StartReact effect critically depends on motor preparation of the requested movement. The prevailing hypothesis states that the prepared motor program may be stored in subcortical structures, from where it can be directly released by the SAS [6,10,11]. However, the mechanism underlying the process of preparing and storing of the motor program remains elusive. The present results provide novel insight by demonstrating that this process does not depend on the recent execution of the requested movement, which possibility could not yet be ruled out on the basis of previous studies.

Moreover, the behavioral advantage of accelerated movement onset with a SAS did not only present itself in the very first trial of the reactive ankle dorsiflexion movement. The accelerating effect of the SAS (relative to the non-startling stimulus) was substantially greater in the first trial when comparing to the subsequent trials. All together, these findings lend further support to the hypothesis that the StartReact phenomenon represents a defensive control mechanism for rapidly and appropriately initiating a movement in response to sensory stimuli signaling a potential threat [4]. Although the involved neural circuitry remains a matter of debate, a recent review has suggested a pivotal role for the pontomedullary reticular formation [4]. This structure not only harbors the large cells that best resemble the neurons involved in very rapid movement initiation in animals, but could also fit with the many (and sometimes seemingly inconsistent) accounts of the StartReact phenomenon in humans that have been published in the past two decades.

Our results also demonstrated that reaction times decreased following the first trial, suggesting an effect of practice on the task at hand. The effects of practice have previously been demonstrated by comparing reaction times of elbow extension between two different measurement days [12]. For trials with a non-startling stimulus the reaction times were shorter on the second measurement day, yet no practice effect was reported for trials with a SAS. The present results concur with these previous findings with respect to the faster reaction times in succeeding compared to the first non-SAS trial. Yet, in contrast with the results of Maslovat and colleagues, we also demonstrated a small but significant reduction in reaction times with a SAS from the first to succeeding trials.

Repetition of movements likely results in lowering of threshold levels for triggering a response [12,13], which may explain why we observed an overall reduction in reaction times from the first to succeeding trials of the same modality. Yet, this reduction was very pronounced from the first to the succeeding trials (see Fig 2), without any evidence for a further gradual acceleration of reaction times across trials. Arguably, with further movement repetitions, no or only limited gains in ankle dorsiflexion reaction times are to be expected, as it involves a low-complexity and highly common movement, which explanation would fit with the absence of further acceleration from the second trial onwards.

Furthermore, we suggest that in addition to potential effects of practice, the delayed onsets in the very first trial could also be due to the novelty of the experimental context or the stimulus to which the participants had to react. Integrating this novel information with the motor memory of the requested movement presumably involves cortical processing. As the SAS-induced release of the requested movement is suggested to bypass the cortex [6,11], this integrative process may not affect reaction times in the very first trials that involve a SAS. This explanation appears to fit with the very large acceleration in reaction times of almost 100 ms that we observed in first trials with a SAS compared to those with a non-startling stimulus. Under this hypothesis, it may also be predicted that in session 2, the familiarity with the experimental context and the imperative stimulus would no longer elicit excessively slow first-trial reaction times in response to non-startling stimuli, which is indeed what we observed. As a result, the SAS-induced gains in reaction times in this trial were similar to those in succeeding trials in session 1.

Importantly, the accelerating effects of the SAS that we observed were not mediated by a startle reflex, as we did not observe differences in the onset latencies of tibialis anterior muscle in the presence or absence of SCM activity. Furthermore, it is also unlikely that the early SAS-induced activity in tibialis anterior in the very first trial could be attributed to a startle reflex, as the onset latencies of ~150 ms were substantially longer than those observed for true startle reflexes in this muscle [7,14,15]. A further argument for the tibialis responses in the first trial not being due to a startle reflex is provided by the finding that the accelerating effects of the SAS also applied to gastrocnemius activation and movement onset. A final observation in support of this interpretation is the discrepant pattern of onset latencies between tibialis and SCM across trials with a SAS. Tibialis onsets became significantly faster from the very first to succeeding trials, while the onsets of startle reflexes in SCM remained constant. Hence, our findings are consistent with the theory that startle reflex and StartReact effects are dissociated [11,16].

The StartReact effect is commonly defined as the accelerated release of a pre-programmed response when a startling stimulus is paired with the imperative stimulus [1,3,11,13], yet there is no consensus on the upper boundary of StartReact reaction times. Although the first-trial reaction times were substantially reduced with a SAS, it must be mentioned that the absolute values were within the range of non-SAS reaction times in succeeding trials. The longer SAS-induced reaction times in the first compared to succeeding trials indicate additional delays in the pathway mediating the rapid release of the movement during the first exposure. The origin of these delays is yet unknown and would be an interesting topic for further research.

Another observation that deserves mention is that the SAS-induced onset latencies in TA in the present study were longer than those observed in our previous study [6]. This discrepancy may be due to the somewhat different experimental setup in the two studies. In the previous study, we used rather intense *visual* warning and imperative stimuli. For inducing the StartReact effect, we added a SAS to the visual stimulus. In contrast, in the present study, we used low-intensity *auditory* warning and imperative stimuli; we replaced the low-intensity stimulus by the SAS for inducing the StartReact effect. Consequently, the reaction times of the two studies may also differ. Previous studies showed that reaction times are shorter with multisensory stimulus compared to single sensory stimulus [17], and the combination of a visual imperative stimulus with a startling acoustic stimulus—as used in our previous study—might therefore result in shorter reaction times compared to an auditory stimulus alone. Another difference between the two studies concerns the method of onset latency detection; in the present study we detected TA onsets on a single trial basis, whereas in our previous study onset detection was based on ensemble average traces [1].

Interestingly, we found that reaction times in the first non-SAS trial following a trial with a SAS were significantly slower than those in subsequent non-SAS trials. Such post-startle slowing has also been documented in a previous study that investigated the effect of startle on action awareness [18], yet the mechanism underlying post-startle slowing remains elusive. It may be that this systematic delay in onset latencies is due to post-surprise slowing—i.e. trials following a surprising stimulus lead to slower reaction times in the following trial [19,20]. The SAS can be regarded as a surprising stimulus as it triggers an involuntary physiological response [21]. The underlying physiological mechanism of post-surprise slowing is unclear, yet it appears that the SAS may have induced a transient increase in threshold levels of neuronal excitability, either in afferent or efferent circuitry.

In conclusion, our study confirms that the StartReact phenomenon indeed applies to movements that are performed without previous practice in the experimental context. The vast SAS-induced movement acceleration in the very first trial suggests that under these circumstances, novel context-specific information may not be integrated with the motor memory for preparing and releasing a motor program. All together, these findings are in line with the hypothesis that StartReact may provide a behavioural advantage in those circumstances that require a fast response to a threatening stimulus; for example, in a situation in which a defensive reaction is needed.
